# Early bactericidal activity of isoniazid: Advances and implications for dosage adjustment

**DOI:** 10.1016/j.bsheal.2026.07.001

**Published:** 2026-07-07

**Authors:** Ningjian Cai, Xiaoyi Jiang, Chao Zhang, Chaonan Xing, Xinchun Chen, Hairong Huang

**Affiliations:** a National Clinical Laboratory on Tuberculosis, Beijing Key Laboratory for Drug-Resistant Tuberculosis Research, Beijing Chest Hospital, Capital Medical University, Beijing Tuberculosis and Thoracic Tumor Institute, Beijing 101149, China; b Guangdong Provincial Key Laboratory of Regional Immunity and Diseases, Department of Pathogen Biology, Shenzhen University Medical School, Shenzhen 518000, China

**Keywords:** Tuberculosis, Isoniazid, Early bactericidal activity, NAT2 genotype, High-dose

## Abstract

Isoniazid (INH) remains a cornerstone of tuberculosis (TB) treatment; however, its early bactericidal activity (EBA) and associated adverse effects vary substantially among individuals. Although personalized dosing has been proposed, its clinical implementation remains limited, largely due to incomplete characterization of the predictive factors. This review synthesizes evidence from PubMed-indexed EBA studies of INH to identify key determinants of its variable efficacy and to guide individualized dose selection across different clinical settings. We found that individual N-acetyltransferase 2 (NAT2) acetylator status is a critical predictor of both the EBA of INH and the risk of drug–induced liver injury (DILI). Geographic variation in NAT2 acetylator phenotypes likely explains a substantial proportion of the regional disparities in reported EBA. Additionally, escalating the INH dosage may overcome low–level resistance associated with *inhA* promoter mutations. In conclusion, personalizing INH dosage based on NAT2 acetylator type—especially for rapid acetylators—and increasing doses to address *inhA*–mediated resistance may improve therapeutic efficacy and safety. Integrating these host and pathogen factors into clinical practice could help optimize TB treatment outcomes across diverse populations and resistance profiles.

## Highlights

• NAT2 polymorphisms drive marked inter-population variation in INH efficacy.
• NAT2-guided INH dosing, especially for rapid acetylators, improves efficacy and safety.
• High-dose INH (15 mg/kg) has modest EBA for *inhA* mutants but is ineffective for *katG*.

## 1 Introduction

Tuberculosis (TB) remains one of the most devastating infectious diseases worldwide, and the emergence of drug resistance has further exacerbated the epidemiological burden. New or repurposed anti-TB drugs are urgently needed to contain this ancient disease. However, evaluating the efficacy of a single anti-TB drug through clinical trials is challenging, because TB treatment necessarily requires multidrug combinations and entails an extremely long treatment course. Consequently, although clinical trials are costly and labor-intensive, their direct outcomes reflect the efficacy of a given regimen rather than that of an individual drug. Alternatively, animal models can be used to evaluate new anti-TB drugs directly, but specialized animal facilities are scarce and expensive to maintain. More importantly, animal models may not fully recapitulate the treatment response in humans. The lack of a feasible and reliable assessment system thus constitutes a major bottleneck in new anti-TB drug development.

The early bactericidal activity (EBA) study is a scientifically rigorous method for evaluating anti-TB drugs and represents a key phase in the drug development continuum [1]. EBA studies are typically conducted by administering the study drug as monotherapy for 2 days (EBA_0__–__2_), 7 days (EBA_0__–__7_), or occasionally 14 days (EBA_0__–__14_). Bacterial load changes are monitored by enumerating colony-forming units (CFU) in patients' sputum samples, collected daily at baseline and during drug administration. In recent years, many studies have measured the time to positivity (TTP) in MGIT960 culture in parallel with conventional CFU-based EBA, thereby providing a more accurate reflection of bacterial load changes in specimens [2], [3]. However, because the majority of EBA studies on isoniazid (INH) were performed in the early period, TTP data are not available for most of these studies.

INH is a cornerstone of first-line TB therapy. By rapidly reducing sputum bacterial load during the initial days of treatment, it decreases transmission risk and helps prevent the emergence of resistance to other first-line agents [4], [5]. In addition, high-dose INH is recommended by the World Health Organization (WHO) as a Group C drug for the treatment of drug–resistant TB.

In 1980, Jindani et al. [6] conducted the first conceptual EBA study, evaluating multiple anti-TB regimens over a 2-day course; in 2003, Jindani et al. [4] extended this approach to test more drug combinations over 14 days. These studies revealed that INH exhibited the strongest bactericidal effect during the early phase of TB treatment, affirming its pivotal role in standard regimens. Although the recommended dosage of INH (5 mg/kg) is well recognized worldwide, recent progress in EBA studies of INH has generated enriched data that may offer new insights into optimal INH dosing.

## 2 Review methodology

This article is a narrative (non-systematic) review designed to synthesize, integrate, and critically evaluate the existing literature on the EBA of INH, with a focus on the role of N-acetyltransferase 2 (NAT2) acetylator status and drug resistance mechanisms. The goal is to provide a conceptual overview, identify key debates, and highlight knowledge gaps to inform future research and clinical translation, rather than to produce a quantitative pooled estimate.

### 2.1 Literature search

A comprehensive search was conducted using combinations of the following keywords: “isoniazid” OR “INH” AND “early bactericidal activity” OR “EBA” AND “N-acetyltransferase 2” OR “NAT2” OR “acetylator status” OR “pharmacogenetics” AND “drug-resistant tuberculosis” OR “*inhA*” OR “katG”. The search covered publications from PubMed database. Only articles published in English were considered. In addition, reference lists of key retrieved articles were manually screened to identify further relevant studies.

### 2.2 Study selection considerations

Given the narrative scope, we did not apply formal systematic review inclusion/exclusion criteria. Instead, we focused on original EBA studies that reported quantitative EBA data for INH in humans (both drug-susceptible and drug-resistant tuberculosis), studies that examined the relationship between NAT2 genotype and INH pharmacokinetics/pharmacodynamics or toxicity, and studies characterizing INH resistance mechanisms (particularly *inhA* and *katG* mutations). Priority was given to seminal early studies, key clinical trials, and recent advances that have shaped the field. We excluded case reports, conference abstracts, non-English articles, and studies where INH was tested only in combination without a monotherapy arm for EBA assessment.

## 3 EBA as a tool for anti-TB drug evaluation

EBA studies serve multiple purposes in anti-tuberculosis drug development. First, they predict drug efficacy, as a significant decrease in bacterial load reflects strong *in vivo* activity of the tested agent. Second, they inform appropriate dosage and administration interval selection by comparing the rate of bacterial load reduction against safety profiles—a crucial step before large-scale clinical trials for novel anti-TB drugs. Third, EBA studies evaluating different drug combinations provide direct efficacy evidence to guide appropriate treatment regimen establishment. Fourth, rapid bacterial load decline suggests prompt elimination of rapidly growing bacteria in pulmonary cavities, thereby indicating the drug's capacity to reduce patient infectivity. Fifth, extended EBA—measuring killing rates between day 2 and day 7 or longer—has been advocated as an early indicator of sterilizing activity and the ability to eliminate slowly replicating, persistent bacilli in tissues. As such, extended EBA is regarded as a potential predictor of long-term treatment outcomes, although this correlation requires further validation.

Beyond its specific applications, a fundamental advantage of EBA studies lies in their feasibility. Due to the mono-drug chemotherapy design, these studies require small sample sizes and short observation periods (typically a few days), making them considerably more practical and cost-effective than full-scale clinical trials. Moreover, EBA outcomes directly reflect the *in vivo* efficacy of a single drug information that cannot be readily obtained from combination regimen trials.

Despite its advantages, the EBA methodology faces several intrinsic challenges that limit reproducibility. Collecting adequate sputum samples at regular intervals is often difficult, particularly in later study stages when patients cannot produce sufficient volumes. The CFU enumeration process is labor-intensive and operator-dependent, hindering standardization across laboratories. Moreover, the slow growth of *Mycobacterium tuberculosis* prolongs incubation periods and increases contamination risks. Certain drugs pose additional timing issues: pyrazinamide lacks activity in the first two days, and bedaquiline shows no obvious efficacy within the first ten days, making standard EBA unsuitable for their objective evaluation [6], [7], [8], [9], [10].

Beyond these technical hurdles, a more fundamental limitation concerns the value of EBA as a surrogate endpoint for long-term clinical outcomes. The commonly reported EBA_0-2_ has never been prospectively validated to predict patient-important endpoints such as sustained culture conversion, relapse-free cure, or survival. The rapid early bacterial decline reflects killing of actively replicating bacilli, but does not capture sterilizing activity against slowly replicating or persistent populations—the latter being critical for relapse prevention. Furthermore, EBA_0-2_ is heavily influenced by baseline sputum bacterial load and patient-specific factors (e.g., pulmonary cavity characteristics), which introduce heterogeneity and limit comparability across studies [11], [12]. Thus, while a positive EBA result indicates on-target bactericidal activity, it should not be overinterpreted as a direct predictor of clinical effectiveness. EBA is most appropriately used as a screening and dose–selection tool in early-phase drug development; definitive evidence for regulatory approval still requires large, randomized controlled trials with long-term follow-up.

Consistent with these concerns, a systematic comparison of the INH EBA studies included in this review reveals substantial heterogeneity across several key dimensions (Table 1). Study populations vary from newly diagnosed, drug-susceptible patients to INH-resistant cases with specific *inhA* or katG mutations. Baseline bacterial load, cavity characteristics, and prior treatment history all known to influence EBA, are rarely standardized. Dosing regimens differ widely (3–20 mg/kg, weight-based vs. fixed doses), as do EBA assessment durations (2 to 14 days) and sputum collection schedules. Endpoint definitions and microbiological methods are also heterogeneous: most studies report log CFU decline, but some use TTP or molecular methods, and EBA is calculated over different intervals (e.g., EBA_0-2_, EBA_2__-__7_). Moreover, NAT2 genotyping, a critical host factor, is absent from many older studies, and classification schemes vary (slow vs. rapid, or three categories), limiting comparability. Given this heterogeneity, a formal meta-analysis is methodologically inappropriate. Qualitative cross-study comparisons must be interpreted with caution, as observed differences may reflect true pharmacodynamic variation, methodological artifacts, or both. To improve future research, we recommend standardized reporting of EBA studies, including detailed patient characteristics, consistent NAT2 classification, and pre-defined primary endpoints, to facilitate meaningful cross-study synthesis and, where appropriate, meta-analysis.

**Table 1:** Summary of key characteristics of included EBA studies on isoniazid.

Study	Population	INH dose	NAT2 genotyped	EBA endpoint (s)	Microbiological method	Sputum collection days	Main EBA result
Jindani et al. (1980) [6]	Newly diagnosed, previously untreated	150 mg 300 mg 600 mg	NA	EBA_0-2_; EBA_2-14_; EBA_0-14_	CFU on solid media	0, 2, 4, 6, 8, 10, 12, 14	INH 300 mg shows strongest EBA_0-2_
Donald et al. (2004) [30]	Newly diagnosed, previously untreated	≤37.5 mg 75 mg 150 mg 300 mg 600 mg	RA; IA; SA	EBA_0-2_	CFU on solid media	0, 2	SA shows higher EBA_0-2_ than IA and RA generally
Katiyar et al. (2008) [42]	INH-resistant	16–18 mg/kg	NA	Sputum conversion rate	CFU on solid media	Up to day 30	2.38 times faster conversion in the high-dose INH group
Dooley et al. (2020) [45]	INH-resistant (katG or *inhA*)	5 mg/kg 10 mg/kg 15 mg/kg	NA	EBA_0-2_; EBA_2-7_	CFU on solid media and TPP	0, 2, 7	High dose INH treatment shows meaningful EBA in the *inhA* mutation but not in katG mutation
Gausi et al. (2024) [46]	INH-resistant (katG or *inhA*)	5 mg/kg 10 mg/kg 15 mg/kg 20 mg/kg	RA; IA; SA	EBA_0-7_	TTP	0, 2, 7	SA/IA with *inhA*: meaningful EBA at 10–15 mg/kg; katG: marginal even at 20 mg/kg

Abbreviations: INH, isoniazid; NAT2, N-acetyltransferase 2; EBA, early bactericidal activity; RA, rapid acetylators; IA, intermediate acetylators; SA, slow acetylators; NA, not available data; CFU, colony-forming unit; TPP, Target Product Profiles; TTP, time to positivity.

## 4 N-acetyltransferase 2 (NAT2) pharmacogenetics as a host determinant of INH EBA

INH inhibits bacilli in a time- and concentration-dependent manner; therefore, the optimal dosage is defined as the maximum safe dose based on the safety profile. The first EBA study with escalated INH dosages, conducted by Jindani et al. [6] provided the initial insights into the appropriate INH dosage. They found that EBA_0__–__2_ was 0.57, 0.72, and 0.48 log_10_ CFU/day when INH was administered at 150 mg/day, 300 mg/day, and 600 mg/day, respectively (Table 2). The 600 mg/day dose yielded paradoxical outcomes, which may be attributable to sampling bias, as only four cases were included. Subsequently, Donald et al. [13] examined the logarithmic relationship between EBA and INH dose and obtained a smooth curve peaking at 300 mg/day (EBA_0__–__2_ of 0.554 log_10_ CFU/day), with the EBA_0_–_2_ at 600 mg/day being similar to that at 300 mg/day (Table 2). These studies offered valuable insights and provided a rationale for the 300 mg/day dosage in the standard first-line anti-TB regimen [14], [15], [16], [17], [18], [19]. However, these early studies had several limitations: it is well recognized that INH metabolism varies considerably among individuals and that body weight significantly affects drug exposure, yet these factors were not accounted for. Moreover, the small sample sizes may have introduced substantial bias.

**Table 2:** The early bactericidal activity of isoniazid.

Study	Dose (mg/day)	Patients (*n*.)	EBA_0-2_ (SD)	95% CI	Side effect
Jindani et al. [6] (Kenya, 1980)	150	4	0.568 (0.29)	NA	
	300	4	0.722 (0.1)	NA	NA
	600	4	0.471 (0.28)	NA	
Chan et al. [28] (China, 1992)	300	14	0.431 (0.37)	NA	NA
Sirgel et al. [14] (America, 1993)	300	11	0.495 (0.19)	NA	No serious adverse events occurred
Donald et al. [13] (South Africa, 1997)	18.75	10	0.111 (0.12)	−0.027 to 0.195	NA
	37.5	10	0.209 (0.18)	0.083 to 0.334	
	75	10	0.316 (0.17)	0.196 to 0.435	
	150	11	0.397 (0.27)	0.214 to 0.579	
	300	12	0.554 (0.21)	0.146 to 0.691	
	600	11	0.539 (0.21)	0.396 to 0.680	
Chambers et al. [15] (America,1998)	300	11	0.600 (0.30)	NA	NA
Sirgel et al. [16] (China, South Africa, Kenya, 2000)	300 18.5 300 18.5 300 18.5	10 13 8 12 8 9	0.371 (0.30) −0.012 (0.19) 0.649 (0.25) 0.094 (0.33) 0.583 (0.29) 0.307 (0.45)	0.160 to 0.580 −0.120 to 0.100 0.430 to 0.870 −0.170 to 0.360 0.350 to 0.820 −0.030 to 0.640	NA
Gosling et al. [17] (Tanzania, 2003)	300	12	0.770 (0.37)	0.540 to 1.000	NA
Johnson et al. [18] (America, 2006)	300	10	0.670 (0.17)	NA	NA
Dietze et al. [19] (Brazil, 2008)	300	10	0.670 (0.35)	0.420 to 0.910	No serious adverse events occurred

The unit of EBA is log_10_ CFU/day. Abbreviations: EBA, early bactericidal activity; *n.*, number; NA, not available data; SD, standard deviation; CI, confidence interval; CFU, colony-forming unit.

INH is primarily metabolized by NAT2 [20], which exhibits large inter-individual variability in acetylating activity [21], [22]. Individuals can be classified as slow acetylators (SA), intermediate acetylators (IA), or rapid acetylators (RA) according to their NAT2 gene polymorphisms. Owing to these genotypic differences, several–fold variations in plasma exposure have been observed in patients receiving the standard INH dose [23]. NAT2 acetylator status profoundly influences INH pharmacokinetics: SA exhibit reduced NAT2 enzyme activity, resulting in impaired INH metabolism, elevated serum drug concentrations, and prolonged drug exposure, thereby conferring an increased risk of toxicity. Conversely, RA and IA demonstrate higher enzyme activity, leading to accelerated drug metabolism [24], [25]. This rapid clearance may prevent INH from reaching effective therapeutic concentrations, potentially leading to treatment failure [26].

Globally, INH therapy is associated with elevated liver enzyme levels in 10%–20% of recipients, with fatal hepatitis estimated at 0.05%–1.00% and peripheral neuropathy affecting 2.0%–6.5% of patients (with higher incidence in the elderly) [27]. In addition to these well-recognized adverse effects, other minor reactions—including constipation, orthostatic hypotension, nocturnal frequent urination, increased dyspnea, and sensory abnormalities—have also been reported during INH treatment. Moreover, more severe adverse reactions, such as neuropsychiatric symptoms (e.g., confusion and seizures) and severe allergic reactions, have been documented and warrant clinical attention.

Although these percentages suggest a relatively low rate of severe adverse events, they represent a substantial absolute number of affected individuals, given the high global burden of tuberculosis. Notably, a *meta*-analysis by Surarak et al. [23] reported that the mean prevalence of drug-induced liver injury (DILI) was significantly higher in the SA group (36.23 ± 19.84%) compared to the IA (19.49% ± 18.20%; *P* < 0.001) and RA (20.47% ± 20.68%; *P* < 0.0001) groups, with the prevalence of elevated liver enzymes also differing significantly across the three groups (*P* = 0.042). These marked differences in drug exposure among acetylator types would inevitably influence both bacillary killing efficacy and toxicity risk; however, early EBA studies did not consider this confounding factor.

Another important issue that needs to be addressed is that the constitution of these three acetylator types are significantly different among regions. According to a systematic review analysing thousands of individuals, SA is the dominant genotype in Africa and accounts for 60.16%, while RA constitutes 18.28%. In contrast, Asia and the Americas have a relatively even distribution of these three genotypes; SA accounts for 34.42% and 41.94%, and RA accounts for 28.54% and 26.81%, respectively [23]. Notably, the heterogeneity of the constitution in different countries from the same continent are common. These evident differences in different ethnicities may explain the significant variations in EBA results across different areas. For instance, studies conducted by Chan et al. [28] and Nie et al. [29] in China reported EBAs of INH at 0.431 and 0.39 log_10_ CFU/day; respectively, under a daily dose of 300 mg, which were lower than those observed in similar studies in Africa and the United States (Table 1). Based on these facts, the standard INH dosage of 300 mg/day might be sufficient for most of the American and African populations, but not for many Asian populations. For the same reason, the conclusion drawn from an EBA study performed in a specific country with a limited number of patients without knowing their acetylator types could not easily be extrapolated to other regions or races.

Donald et al. [30] conducted the first EBA study of INH that considered acetylator genotypes. In that study, SAs achieved the highest EBA, which was significantly greater than that in the IA and RA groups; however, no significant difference was observed between the IA and RA groups (Table 3). Additionally, Donald et al. [30] concluded that 300 mg/day INH is insufficient for RA patients, a finding subsequently supported by other studies [31]. Therefore, timely identifying the NAT2 acetylator types of patients before launching an anti-TB treatment would be crucial to adjust the dosage of INH.

**Table 3:** Influence of NAT2 acetylator types on EBA of isoniazid.

Dose (mg)	Group RA		Group IA		Group SA	
	Patients (*n.*)	Mean EBA	Patients (*n.*)	Mean EBA	Patients (*n.*)	Mean EBA

75	1	0.298	9	0.290	9	0.379
150	3	0.532	9	0.476	4	0.824
300	10	0.523	5	0.467	6	0.606
600	5	0.325	5	0.695	5	0.686

The unit of EBA is log_10_ CFU/day. Abbreviations: NAT2, N-acetyltransferase 2; EBA, early bactericidal activity; RA, rapid acetylators; IA, intermediate acetylators; SA, slow acetylators; *n.*, number; CFU, colony-forming unit.

## 5 Clinical implications of NAT2 genotype-guided dosing in INH therapy

Previous findings indicate that a genetic polymorphism is associated with large interindividual differences in EBA of INH which may lead to therapy failure. However, how to translate the finding of the EBA studies still needs to be determined.

Azuma et al. [32] demonstrated the therapeutic benefit of tailoring INH doses based on NAT2 genotype during the intensive phase of the standard six‐month, four‐drug regimen for new pulmonary TB cases. In the genotype-guided treatment (PG-treatment) group, SA received a lower dose INH experienced no INH-induced liver injury and comparable clinical improvement when compared with the standard dosage group. Conversely, the RA in the PG-treatment received higher dose of INH, achieving a 25% greater rate of clinical improvement with less than 5% experiencing INH-related liver injury. Accumulating evidence has revealed that under the four-drug standard regimen, patients with low INH plasma concentrations have a propensity for treatment failure or early relapse of TB [33], [34]. Specifically; Azuma et al. [32] identified that RA was a major risk factor of treatment failure. A recent study by Kasamatsu et al. [35] revealed that RA genotype was associated with increased all-cause mortality within 1 year of starting an INH-containing regimen and the risk was even greater in patients with INH-resistant TB; with RA individuals exhibiting a 4.68-fold higher risk compared to those with IA. These studies suggest that inadequate treatment in RA patients can negatively affect both short-term and long-term outcomes, and underscore the necessity of NAT2 genotype-guided INH dosing. Unfortunately, up to now, the optimal dosage for different acetylator types remains uncertain due to limited studies. Jing et al. [36] established the first population pharmacokinetic model of INH in Chinese TB patients. Their model revealed that 98% of RA patients and 89.1% of IA patients could not reach an effective drug serum concentration when using the standard 300 mg/day INH dose. Based on dose simulations that integrated efficacy and safety considerations, the authors proposed hypothetical optimal daily doses of 800 mg/day for RA, 500 mg/day for IA, and 300 mg/day for SA patients. While the evidence synthesized in this review suggests that NAT2 genotype–guided isoniazid dosing holds promise for improving treatment outcomes, it is important to emphasize that most of the supporting data come from observational studies, pharmacokinetic/pharmacodynamic modeling, or small–scale clinical trials with limited sample sizes. Large, prospective, randomized controlled trials validating the proposed dosing algorithms (e.g., 800  mg/day for RA, 500  mg/day for IA) are currently lacking. Furthermore, key populations—including children, pregnant women, HIV–coinfected individuals, and malnourished patients—remain underrepresented in the available literature. Safety data, particularly regarding neurotoxicity and hepatotoxicity at higher doses, also require further characterization across diverse populations. Therefore, while NAT2–guided dosing is a promising strategy, it should be considered hypothesis–generating rather than evidence–based guidance. Prospective validation in well–designed, adequately powered clinical trials is essential before any routine clinical implementation can be recommended.

## 6 EBA study in high-dose INH evaluation for INH-resistant TB

Drug-resistant TB is a big threat globally, while INH resistance is one of the most common resistant types, and approximately 10% of the global *M. tuberculosis* isolates are resistant to INH [37]. INH is a prodrug that requires activation by the bacterial catalase–peroxidase enzyme *katG*. Once activated, INH forms a complex with NAD+, which inhibits *inhA* (enoyl-ACP reductase), thereby blocking mycolic acid synthesis and leading to cell wall defects. The major mechanisms of INH resistance involve mutations in the *katG* gene particularly the S315T mutation which impair drug activation, and mutations in the promoter region of the *inhA* gene (e.g. − 15C to − 15 T and − 8T to − 8C), which cause overexpression of the target enzyme and reduce or prevent the formation of the INH–NAD adducts required for bactericidal activity [38], [39]. Mutations in *inhA* and *katG* result in varying degrees of resistance to INH, with *katG* mutations typically conferring a higher level of resistance [40]. *In vitro* studies show that the minimum inhibitory concentration of INH for susceptible strains ranges from 0.03 to 0.06 mg/L. In contrast, the MIC for strains with *inhA* mutations, *katG* mutations, and *inhA* and *katG* double mutations are 0.21 mg/L, 2–10 mg/L, and ≥ 12 mg/L, respectively [41]. These facts suggest that increasing the INH dose could potentially overcome reduced therapeutic efficacy due to low-level INH resistance.

Early clinical evidence suggested that high-dose INH may offer benefits in drug-resistant tuberculosis, although the underlying mechanisms were not fully elucidated. A landmark clinical trial by Katiyar et al. demonstrated that high-dose INH (16–18 mg/kg) led to sputum conversion 2.38 times faster in INH-resistant patients compared with those who did not receive INH [42]. Several other clinical studies have also indicated that high-dose INH may improve treatment outcomes in drug-resistant TB, although its overall effectiveness remains uncertain and continues to be debated [42], [43]. Subsequent studies have refined our understanding by distinguishing between different resistance mutations. A pivotal clinical trial by Dooley et al. demonstrated that high-dose (10–15 mg/kg) INH monotherapy exhibited significant bactericidal activity against strains harboring *inhA* mutations. Daily administration of 10–15 mg/kg INH manifested EBA against *inhA* mutant strains comparable to that of 5 mg/kg INH against susceptible strains (Table 4) [45]. However, this study did not investigate the influence of the NAT2 genotype. For *katG* mutant strains, only doses exceeding 20 mg/kg achieved meaningful EBA [46].

**Table 4:** EBA in participants with INH susceptible TB and INH*-*resistant TB with *inhA* mutation.

	Controls	Isoniazid resistance by *inhA*		
Dose	5 mg/kg	5 mg/kg	10 mg/kg	15 mg/kg
*n.*	13	13	12	15
EBA_0-2_ IQR	0.40 (0.21, 0.67)	0.09 (−0.01, 0.38)	0.06 (−0.13, 0.39)	−0.09 (−0.55, 0.48)
EBA_0-7_ IQR	0.15 (0.11, 0.25)	0.08 (0.01, 0.14)	0.12 (0.10, 0.23)	0.20 (0.11, 0.28)
EBA_2-7_ IQR	0.09 (−0.43,0.18)	−0.04 (−0.16, 0.02)	0.15 (0.04, 0.29)	0.25 (0.04, 0.48)

The unit of EBA is log_10_ CFU/day. Abbreviations: EBA, early bactericidal activity; INH, isoniazid; *n.*, number; IQR, interquartile range; CFU, colony-forming unit.

More recently, the interaction between host NAT2 genotype and pathogen resistance mutations has begun to be elucidated. Gausi et al. [46] assessed the influence of NAT2 genotype on INH efficacy in drug-resistant TB and found that acetylator status significantly modulated treatment response. Among patients with *inhA*-mutated TB, INH at 10 mg/kg in SA and 15 mg/kg in IA yielded meaningful EBA comparable to 5 mg/kg in participants with susceptible TB. However, for *katG*-mutated cases, even at 20 mg/kg, only SA showed marginal EBA.

These findings have important clinical implications. Given the neurotoxicity and hepatotoxicity risks associated with high-dose INH (particularly in SA), this treatment strategy may offer limited clinical benefit for patients infected with *katG*-mutant strains. As *katG* mutations represent the most prevalent form of INH resistance globally, rapid identification of both the bacterial resistance mutation and the patient's NAT2 genotype is essential before considering high-dose INH therapy.

An important but often overlooked aspect in the evaluation of high-dose INH strategies is the broader interaction between host NAT2 genotype and the risk of acquiring drug resistance. Accumulating evidence suggests that NAT2 acetylator status not only influences treatment efficacy and toxicity but may also modulate the risk of developing drug-resistant TB. A Peruvian study noted that the fast metabolizer phenotype could increase the risk of acquiring INH resistance, thereby elevating the risk of multidrug-resistant TB (MDR-TB) or treatment failure [47]. Mechanistically, the link between NAT2 genotype and resistance can be explained by drug exposure dynamics. RA achieve lower serum INH concentrations, which may fail to eradicate susceptible bacterial populations, thereby exerting selective pressure for the emergence of resistant mutants [47], [48], [49]. Furthermore, a study by Unissa et al. [48] had suggested that RA variants of human NAT2 lead to faster inactivation of INH, potentially serving as a host risk factor for the development of INH resistance.

These converging lines of evidence highlight that host and pathogen factors are not independent in determining treatment outcomes. When considering high-dose INH strategies to overcome low-level resistance (e.g., *inhA* mutations), the patient's NAT2 genotype becomes a crucial influencing factor. A dose that is sufficient for a SA with an *inhA* mutation may be sub-optimal for a RA carrying the same resistant strain. Therefore, future clinical trials evaluating high-dose INH for drug-resistant TB should stratify patients by both NAT2 genotype and resistance mutation type to establish evidence-based, personalized dosing algorithms.

However, high-dose INH (≥10  mg/kg) is associated with dose-dependent increases in hepatotoxicity and neurotoxicity (peripheral neuropathy, seizures). SA are at particularly high risk because they achieve higher serum INH concentrations at any given dose. In clinical practice, the safety margin of high-dose INH has not been established in large, diverse populations, and prolonged use beyond the initial EBA period has not been systematically studied.

Outcome data are limited to short-term surrogates. Most evidence supporting high-dose INH comes from EBA studies measuring bacterial load decline over 7–14 days. Whether this translates into improved long-term outcomes such as sustained sputum culture conversion, relapse-free cure, or reduced mortality remains unknown. No large, randomized controlled trial has compared high-dose INH-containing regimens versus standard regimens for *inhA*-mutant tuberculosis with long-term follow-up.

## 7 EBA of anti-TB regimens containing INH to assess the appropriate drug combinations

Several EBA studies comparing regimens containing INH have consistently found that adding other drugs does not significantly improve EBA over the first two days (Table 5) [50], [51], [52], [53]. This lack of additional benefit may be caused by INH harboring much superior antibacterial activity in the first two days of treatment, which overshadowed the effects of the other drugs. However, over a longer period (EBA_0-14_), the combination of INH with other drugs, such as RMP, significantly increased the EBA, indicating that these additional drugs play a major role later in the treatment course. During the initial two days, a large number of replication-active *M. tuberculosis* are present, making INH particularly effective. Once these active bacilli are eliminated, the effect of other drugs emerges gradually against the remaining bacteria, which may be in a quiescent or dormant state within the lesions. This hypothesis echoed the multiple-drug combination strategy of anti-TB treatment.

**Table 5:** EBA in drug combinations containing INH.

Study	Combinations	Patients (*n*.)	EBA_0-2_	EBA_0-14_	Side effect
Jindani et al. (1980) [6]	H	4	0.722	0.192	NA
	HR	4	0.714	0.287	
	HE	4	0.702	0.140	
	HZ	4	0.494	0.155	
	HS	4	0.510	0.123	
	HRE	4	0.487	0.178	
	HRS	4	0.321	0.168	
	HSZ	4	0.799	0.246	
	HSRZ	4	0.685	0.232	
	HSRZE	4	0.686	0.273	NA
Botha et al. (1996) [50]	HRZ	9	0.250	NA	NA
Diacon et al. (2013) [9]	HRZE	8	0.413	0.112	No serious adverse events occurred
Wallis et al. (2014) [51]	HRZE	9	0.537	NA	No serious adverse events occurred
Furin et al. (2016) [52]	HRZE	14	0.424	0.185	No serious adverse events occurred
Dawson et al. (2023) [53]	HRZE	16	NA	0.199	No serious adverse events occurred

The unit of EBA is log_10_ CFU/day. Abbreviations: H, isoniazid；R, Rifampin；S, Streptomycin；Z, Pyrazinamide；E, Ethambutol; EBA, early bactericidal activity; NA, not available data; *n.*, number.

## 8 Limitations of the narrative approach

This review has inherent limitations compared to a systematic review. The absence of a pre-registered protocol, a comprehensive search of multiple databases (only PubMed was used), and formal inclusion/exclusion criteria may introduce selection bias. Furthermore, the lack of meta-analysis precludes quantitative synthesis of effect sizes. However, the narrative format is appropriate for this topic because the substantial heterogeneity in study designs, populations, dosing regimens, and endpoints makes a formal meta-analysis methodologically unsound. Our conclusions should therefore be interpreted as hypothesis-generating synthesis rather than definitive quantitative evidence.

## 9 Summary

In summary, this review synthesizes decades of EBA studies to propose a refined, evidence-based framework for INH dosing in tuberculosis treatment. We conclude that EBA is not merely a preclinical pharmacodynamic measure but a clinically translatable biomarker that can directly inform dose individualization, bridging the gap between early-phase trials and clinical practice. Based on this premise, we draw two key conclusions with direct clinical implications. First, adjusting INH dosage according to NAT2 acetylator status is viable to optimize treatment outcomes, balancing efficacy against the risk of drug-induced liver injury. Second, higher doses of INH appear practicable for overcoming low-level resistance conferred by *inhA* mutations, offering a potential strategy for managing drug-resistant strains. A central contribution of our review is the integration of both host (NAT2 acetylator status) and pathogen (*inhA* resistance) factors within a unified dosing framework. We argue that optimal INH therapy must consider both dimensions simultaneously—tailoring doses to acetylator type for safety and efficacy in most patients, while exploring high-dose strategies for cases involving *inhA*-mediated resistance. We acknowledge that while EBA, particularly EBA_0-2_, remains a valuable measure of early drug activity, its correlation with long-term outcomes such as relapse and cure is not firmly established and remains an area of active investigation. This limitation is especially pertinent in understudied populations, including children and individuals with HIV coinfection. An additional barrier to clinical implementation is the current lack of a convenient NAT2 genotyping method suitable for routine practice. Most available studies have relied on gene sequencing, which is not readily accessible in clinical laboratories, particularly in resource-limited settings. This further complicates the translation of NAT2-guided dosing strategies into real-world settings. However, several practical strategies could help overcome these barriers, including point-of-care cartridge-based genotyping, integration with existing platforms such as GeneXpert, use of dried blood spots for centralized testing, low-cost phenotyping (e.g., urinary metabolic ratio), and task-sharing with trained non-specialist health workers. These approaches, once validated, may enable feasible and equitable implementation of NAT2-guided isoniazid dosing even in high-burden, resource-constrained environments.

Future studies integrating EBA data with long-term, multicenter clinical follow-up will be essential to fully validate the dose individualization approaches proposed in this review and to address the practical challenges of implementation in diverse populations.
